# Case report of antepartum and postpartum spontaneous coronary artery dissection in a high-risk pregnancy

**DOI:** 10.1093/ehjcr/ytag196

**Published:** 2026-03-12

**Authors:** Andrii Labchuk, Viktoriya Bikeyeva, Giovanni Paolella, Katarzyna Mikrut

**Affiliations:** Division of Cardiology, Lutheran General Hospital, 1775 W Depster Street, Park Ridge, IL 60068, USA; Department of Internal Medicine, Lutheran General Hospital, 1775 W Dempster Street, Park Ridge, IL 60068, USA; Department of Internal Medicine, Lutheran General Hospital, 1775 W Dempster Street, Park Ridge, IL 60068, USA; Division of Cardiology, Lutheran General Hospital, 1775 W Depster Street, Park Ridge, IL 60068, USA

**Keywords:** Pregnancy, Spontaneous coronary artery dissection, Case report, Cardio-obstetrics, Conservative management

## Abstract

**Background:**

Spontaneous coronary artery dissection (SCAD) is a rare, non-atherosclerotic cause of acute coronary syndrome (ACS) that mainly affects women, particularly during or after pregnancy.

**Case summary:**

A 38-year-old woman conceived via *in vitro* fertilization presented at 34 weeks’ gestation with chest pain and rising troponin levels (191 ng/L → 5500 ng/L; reference <52 ng/L). Initially patient was elected for medical management. Day later telemetry detected frequent non-sustained ventricular tachycardia, considered a high-risk feature for underlying SCAD. Coronary angiography demonstrated a Type II SCAD of the mid-LAD (≈50% stenosis, TIMI III flow). She was managed conservatively with aspirin and labetalol. Hours later, she developed pre-eclampsia and underwent urgent caesarean section with complete symptom resolution. Two weeks postpartum, she re-presented with an inferior STEMI due to a Type II SCAD of the right PDA (TIMI II flow). Conservative management with temporary intra-aortic balloon pump support led to full recovery and preserved left-ventricular function.

**Discussion:**

This biphasic case highlights SCAD’s propensity for recurrence in the peripartum period and supports conservative management when coronary flow is maintained. Reference to the 2025 ESC Guidelines for Acute Coronary Syndromes underscores the role of individualized, multidisciplinary care.

Learning points

- **Pregnancy-associated spontaneous coronary artery dissection (P-SCAD)** should always be considered in pregnant and postpartum women presenting with acute chest pain, even without traditional cardiovascular risk factors.
- **Conservative, multidisciplinary management** guided by European Society of Cardiology recommendations often leads to excellent outcomes when coronary flow is preserved.

## Introduction

Spontaneous coronary artery dissection (SCAD) is an uncommon but important non-atherosclerotic cause of acute coronary syndrome (ACS), responsible for up to 40% of pregnancy-related myocardial infarctions. It typically occurs in late pregnancy or the early postpartum period due to hormonal, haemodynamics, and inflammatory vascular changes. Awareness of this entity is critical for early recognition and guideline-directed management, as per the 2025 European Society of Cardiology (ESC) Guidelines for Acute Coronary Syndromes, which recommend a conservative approach when coronary flow is preserved.^1^

## Summary figure

Timeline of clinical events and management showing antepartum SCAD, caesarean delivery, postpartum recurrence, and recovery.

**Figure:**
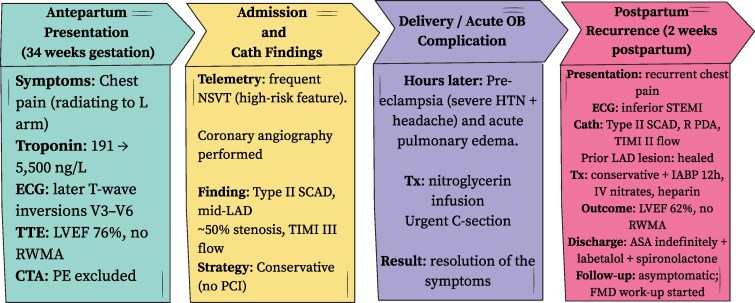
Summary timeline of antepartum and postpartum spontaneous coronary artery dissection (SCAD). Timeline showing antepartum Type II SCAD of the mid–left anterior descending artery managed conservatively, followed by postpartum recurrence involving the right posterior descending artery and successful conservative management with pre-served ventricular function.

## Case presentation

A 38-year-old woman (body mass index 51 kg/m^2^) conceived through *in vitro* fertilization presented at 34 weeks’ gestation with acute, pressure-like substernal chest pain radiating to the left arm. She had no prior cardiac history. Initial ECG was unremarkable; serial high-sensitivity troponins increased from 191 ng/L (reference <52 ng/L) to 5500 ng/L. Repeat ECG showed T-wave inversions in V3–V6.

CTA excluded pulmonary embolism. Transthoracic echocardiography revealed an ejection fraction of 76% without regional wall-motion abnormalities. After multidisciplinary discussion between cardio-obstetrics, MFM (material foetal medicine) and obstetrics team’s decision was made to manage patient conservatively. At that time level of suspicious for SCAD was high but patient was haemodynamically stable without high-risk SCAD features.

Next day after admission telemetry monitoring demonstrated frequent short runs of non-sustained ventricular tachycardia (NSVT), which were considered a potential high-risk feature of underlying SCAD. Given these findings and the rising cardiac biomarkers, coronary angiography was performed and revealed a Type II dissection of the mid-LAD (≈50% stenosis, TIMI III flow). No intervention was undertaken. She was treated with aspirin, labetalol, and nifedipine.

Hours later, she developed severe hypertension and headache consistent with pre-eclampsia. A nitroglycerin infusion was initiated, and an urgent caesarean section was performed. Post-delivery, her chest pain resolved, and she was discharged on medical therapy.

Two weeks postpartum, the patient re-presented with recurrent chest pain and an inferior STEMI pattern on ECG. Repeat angiography demonstrated a long Type II SCAD in the right PDA (TIMI II flow) with complete healing of the prior LAD lesion. Due to difficulty advancing a wire for intravascular imaging and preserved flow, a conservative strategy was chosen with intra-aortic balloon pump support for 12 h, intravenous nitrates, and heparin.

Follow-up echocardiography showed a normal LVEF 62% and no wall-motion abnormalities. She was discharged on aspirin indefinitely, labetalol 300 mg daily, and spironolactone.

At 2-month follow-up, she remained asymptomatic with controlled blood pressure. A work-up for fibromuscular dysplasia was initiated.

## Discussion

Pregnancy-associated SCAD (P-SCAD) presents unique diagnostic and therapeutic challenges. Hormonal and haemodynamics changes of late pregnancy increase vascular fragility, predisposing to intimal tears and intramural haematoma formation.^2^

According to the 2025 ESC Guidelines for the Management of Cardiovascular Disease During Pregnancy, spontaneous coronary artery dissection (SCAD) is the leading cause of acute coronary syndrome in pregnant and postpartum women, accounting for about 43% of pregnancy-related ACS cases, most of which occur within the first week postpartum.^3^ The guideline notes that hormonal, haemodynamics, and inflammatory changes during late gestation and early puerperium increase arterial wall fragility, often in women without traditional cardiovascular risk factors. Hypertensive disorders of pregnancy, multiparity, and pre-eclampsia are recognized predisposing factors. The ESC recommends that ACS, including SCAD, be promptly excluded in any pregnant or postpartum woman with chest pain, using standard diagnostic algorithms.^4^ When coronary flow is preserved, a conservative approach with beta-blockers and single antiplatelet therapy is advised, reserving revascularization for refractory ischaemia or haemodynamics compromise. Multidisciplinary care and postpartum follow-up are essential to improve maternal outcomes and reduce recurrence risk.

This case demonstrates:

- **Biphasic SCAD** affecting different coronary territories during pregnancy and postpartum.
- **Frequent NSVT as a concerning feature** prompting angiography, which confirmed SCAD with preserved flow.
- **Multidisciplinary coordination** between cardiology, maternal-foetal medicine, and critical care leading to excellent outcome.
- **Conservative treatment success**, consistent with ESC recommendations favouring medical therapy when flow is adequate.

Clinicians should maintain a high index of suspicion for SCAD in pregnant or postpartum women presenting with acute chest pain, elevated troponins, or arrhythmias—even without traditional cardiovascular risk factors. Early recognition prevents unnecessary invasive procedures and guides safe, conservative management. Awareness of SCAD’s angiographic patterns, recurrence risk, and supportive management principles is essential for all healthcare providers involved in cardio-obstetric care.^5,6^

According to the 2025 ESC ACS Guidelines, revascularization should be reserved for ongoing ischaemia, haemodynamics compromise, or left main involvement; otherwise, conservative therapy with beta-blockers and single antiplatelet treatment remains standard.

## Patient perspective

Following recovery, the patient expressed relief at the outcome and gratitude for coordinated care. She was counselled on recurrence risk and advised to avoid future pregnancies however patient expressed desire to conceive again. Follow up ongoing.

## Data Availability

Data available on request. The data underlying this article will be shared on reasonable request to the corresponding author.

## Lead author biography

Dr. Andrii Labchuk, MD, is a cardiology fellow at Advocate Lutheran General Hospital in Park Ridge, Illinois, USA.With an early background in midwifery, he developed a deep commitment to women’s health that now shapes his focus in cardio-obstetrics, lipidology, preventive cardiology, and cardio-oncology. Dr. Labchuk is passionate about bridging maternal care and cardiovascular medicine to improve outcomes for women before, during, and after pregnancy. He aims to advance global awareness, education, and prevention strategies in heart disease through multidisciplinary collaboration and patient-centred care.

## References

[1] De Backer J, Haugaa KH, Hasselberg NE, de Hosson M, Brida M, Castelletti S, et al 2025 ESC guidelines for the management of cardiovascular disease and pregnancy: developed by the task force on the management of cardiovascular disease and pregnancy of the European Society of Cardiology (ESC). Eur Heart J 2025;46:4462–4568 40878294 10.1093/eurheartj/ehaf193

[2] Yang C, Chai J, Saw J. Contemporary diagnosis and management of spontaneous coronary artery dissection. Heart 2025;111:1184–1190. 40451276 10.1136/heartjnl-2024-324732

[3] Isogai T, Saad AM, Ahuja KR, Gad MM, Shekhar S, Abdelfattah OM, et al Factors associated with revascularization in women with spontaneous coronary artery dissection and acute myocardial infarction. Am J Cardiol 2022;166:1–8. 34949472 10.1016/j.amjcard.2021.11.024

[4] Hayes SN, Kim ESH, Saw J, Adlam D, Arslanian-Engoren C, Economy KE, et al Spontaneous coronary artery dissection: current state of the science: a scientific statement from the American Heart Association. Circulation 2018;137:e523–e557. 29472380 10.1161/CIR.0000000000000564 PMC5957087

[5] Lawton JS, Tamis-Holland JE, Bangalore S, Bates ER, Beckie TM, Bischoff JM, et al 2021 ACC/AHA/SCAI guideline for coronary artery revascularization. J Am Coll Cardiol 2022;79:e21–e129. 34895950 10.1016/j.jacc.2021.09.006

[6] Egidy Assenza G, Dimopoulos K, Budts W, Donti A, Economy KE, Gargiulo GD, et al Management of acute cardiovascular complications in pregnancy. Eur Heart J 2021;42:4224–4240. 34405872 10.1093/eurheartj/ehab546

